# HnRNPA1 Specifically Recognizes the Base of Nucleotide at the Loop of RNA G-Quadruplex

**DOI:** 10.3390/molecules23010237

**Published:** 2018-01-22

**Authors:** Xiao Liu, Yan Xu

**Affiliations:** Division of Chemistry, Department of Medical Sciences, Faculty of Medicine, University of Miyazaki, 5200 Kihara, Kiyotake, Miyazaki 889-1692, Japan; kyou_ryu@med.miyazaki-u.ac.jp

**Keywords:** hnRNPA1, RNA G-quadruplex, telomere RNA, loop of RNA G-quadruplex, base

## Abstract

Human telomere RNA performs various cellular functions, such as telomere length regulation, heterochromatin formation, and end protection. We recently demonstrated that the loops in the RNA G-quadruplex are important in the interaction of telomere RNA with heterogeneous nuclear ribonucleoprotein A1 (hnRNPA1). Here, we report on a detailed analysis of hnRNPA1 binding to telomere RNA G-quadruplexes with a group of loop variants using an electrophoretic mobility shift assay (EMSA) and circular dichroism (CD) spectroscopy. We found that the hnRNPA1 binds to RNA G-quadruplexes with the 2’-O-methyl and DNA loops, but fails to bind with the abasic RNA and DNA loops. These results suggested that hnRNPA1 binds to the loop of the RNA G-quadruplex by recognizing the base of the loop’s nucleotides. The observation provides the first evidence that the base of the loop’s nucleotides is a key factor for hnRNPA1 specifically recognizing the RNA G-quadruplex.

## 1. Introduction

Human telomeric RNA has been reported to form G-quadruplex structures [1,2,3,4,5,6,7,8,9,10]. We have demonstrated that telomeric RNA G-quadruplex structures play an important role in providing a protective structure to the telomere ends [11]. A previous study has suggested that telomere RNA and heterogeneous nuclear ribonucleoprotein A1 (hnRNPA1) act together to facilitate telomere capping [12]. Moreover, our recent study has demonstrated that the loops present in the telomere RNA G-quadruplex are required for its binding to hnRNPA1 [13]. To further reveal which part of the nucleotides on the loop is recognized by hnRNPA1, here we have prepared a series of RNA with the modified loop (Table 1) and performed an electrophoretic mobility shift assay (EMSA) to examine hnRNPA1 binding to these RNA G-quadruplex oligonucleotides. We have found that the base at the loop of the RNA G-quadruplex plays a key role in the binding of hnRNPA1 to the RNA G-quadruplex.

## 2. Results and Discussion

**Figure 1:**
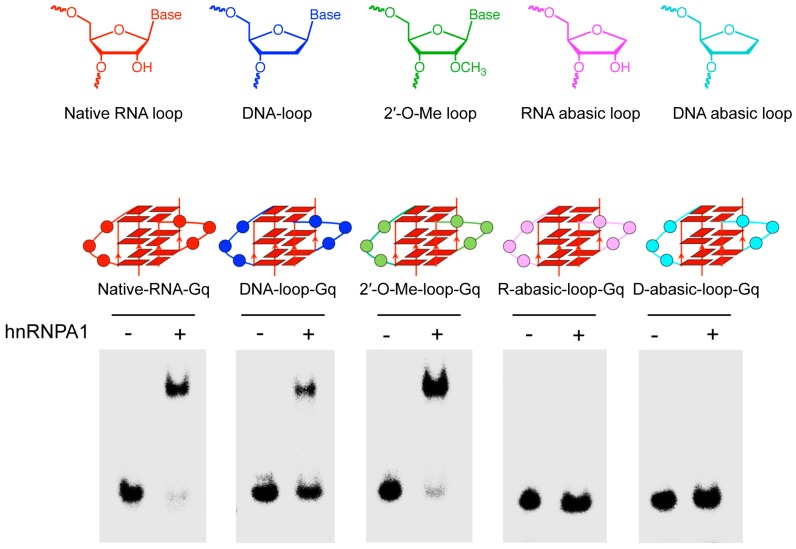
Effect of base of nucleotide at the loop of G-quadruplex RNA on the binding of heterogeneous nuclear ribonucleoprotein A1 (hnRNPA1). Electrophoretic mobility shift assay (EMSA) was performed with hnRNPA1 and ^32^P-labeled RNAs using 8% PAGE in 1 × TBE buffer with 20 mM NaCl in 4 °C for 2 h (80 V). The structures of DNA loop (DNA-loop-Gq), abasic RNA loop (D-abasic-loop-Gq), abasic DNA loop (R-abasic-loop-Gq) and 2’-O-methylribonucleotide loop (2′-O-Me-loop-Gq) are indicated at upper.

We first examined the role of 2′-OH in the loop’s nucleotide on hnRNPA1 binding to the G-quadruplex using a G-quadruplex bearing the DNA loop (DNA-loop-Gq), which lacked the 2′-OH group in the loop (Figure 1). We observed the complex of hnRNPA1 binding to DNA-loop-Gq, which indicated that hnRNPA1 binds to the G-quadruplex with the RNA loop, but does not depend on 2′-OH in the loop’s nucleotide of the RNA G-quadruplex. We next used the loop with the 2′-O-methyl ribonucleotide (2′-O-Me-loop-Gq), which was the replacement of the 2′-OH in the RNA loop, to examine hnRNPA1 binding to the G-quadruplex (Figure 1). The EMSA showed that hnRNPA1 binds to the G-quadruplex with the 2′-O-methyl loop as well as natural RNA (Native-RNA-Gq). The fact that hnRNPA1 does not recognized the 2′-OH on ribose suggests that the base but not the ribose in the loop is responsible for the recognition. To identify the role of the base at the loop for hnRNPA1 binding to the G-quadruplex, we investigated hnRNPA1 binding to the G-quadruplex with a RNA or DNA abasic loop (R-abasic-loop-Gq or D-abasic-loop-Gq), which lacked the base group in the loop (Figure 1). As expected, both the abasic RNA and DNA loops were unfavorable for binding. These results indicate the base at the loop of the RNA G quadruplex plays a key role for hnRNPA1 binding.

**Figure 2:**
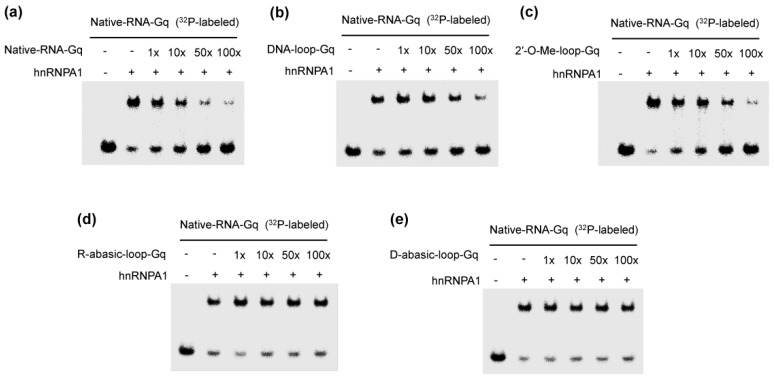
Competitive binding of the complex of heterogeneous nuclear ribonucleoprotein A1 (hnRNPA1) and G-quadruplex with various oligonucleotides: natural telomere RNA (Native-RNA-Gq) (**a**); DNA loop (DNA-loop-Gq) (**b**); 2′-O-methyl ribonucleotide (2′-O-Me-loop-Gq) (**c**); the abasic RNA loop (R-abasic-loop-Gq) (**d**); and the abasic DNA loop (D-abasic-loop-Gq) (**e**). Ratios indicated on the upper. RNA-protein complexes were resolved by 8% polyacrylamide gel electrophoresis and visualized by autoradiography.

We further performed a competitive experiment to evaluate the effect of the base by the addition of the excess an unlabeled competitor (no ^32^P-labeled RNA) (1–100-fold) (Figure 2). Predictably, we observed the clear reduction of hnRNPA1 binding to the G-quadruplex with an addition of excess natural telomere RNA (Native-RNA-Gq) (Figure 2a). The reduction was also observed by addition of a 100-fold excess of DNA-loop-Gq and 2′-O-Me-loop-Gq (Figure 2b,c). On the contrary, the abasic loop in the G-quadruplex of the R-abasic-loop-Gq and D-abasic-loop-Gq had no effect, even at a 100-fold excess (Figure 2d,e). These observations confirmed that hnRNPA1 preferentially recognized the base of the loop in the G-quadruplex. To our knowledge, this is the first report of the protein molecule recognizing the base of the loop in the G-quadruplex.

**Figure 3:**
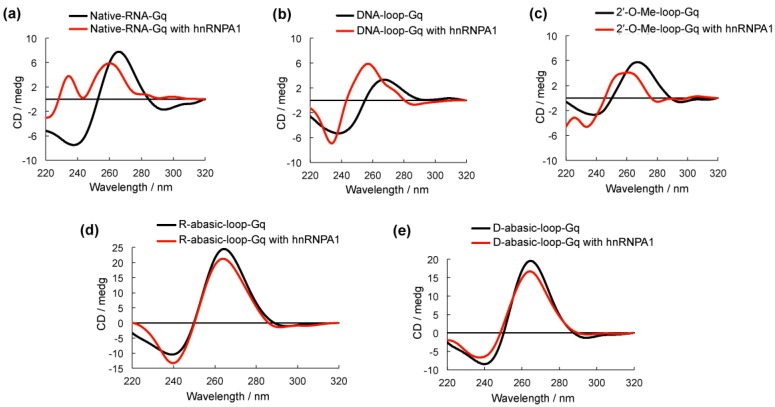
Circular dichroism (CD) spectra of G-quadruplexes with (red line) and without (black line) heterogeneous nuclear ribonucleoprotein A1 (hnRNPA1) in 100 mM NaCl and 10 mM Tris-HCl buffer (pH 7.0). The 5 µM hnRNPA1 was added to 5 µM oligonucleotides and incubated for 1 h at room temperature before spectra were obtained at 20 °C.

Circular dichroism (CD) spectroscopy was used to examine the G-quadruplex structure formed by all of the oligonucleotides [14,15] (Figure 3). DNA-loop-Gq, 2′-O-Me-loop-G and Native-RNA-Gq had a negative peak at approximately 240 nm and a positive peak at approximately 265 nm, which could be assigned to parallel strand G-quadruplex structures (Figure 3a–c). The addition of hnRNPA1 to these G-quadruplexes caused a shift to 260 nm but no significant change in the spectra structure in comparison to these RNA G-quadruplexes alone, suggesting that hnRNPA1 is favorable in binding to the base group of the RNA G-quadruplex. R-abasic-loop-Gq and D-abasic-loop-Gq were identified as the parallel G-quadruplex; however, the addition of hnRNPA1 caused only a slight decrease in the intensity of the positive peak and no shift (Figure 3d,e). We also performed CD melting experiments to examine the stability of the modified oligoribonucleotides (Figure S1 in supplementary materials). The melting profiles of the modified oligoribonucleotides indicated a stable structure compared to the native RNA G-quadruplex, suggesting that the modification on the loop did not influence the conformation of the G-quadruplex structure. These results are consistent with the EMSA results that suggested the RNA G-quadruplexes that lacked the base group in the loop were unfavorable for binding. These results indicate the base in the loop of the RNA G-quadruplex plays a key role for hnRNPA1 binding.

The introduction of abasic sites into the G-quadruplex is a powerful tool to detect the key part of the G-quadruplex for the interaction between the protein and G-quadruplex. In this study, we used an intermolecular dimeric G-quadruplex with two equivalent loops. Alternatively, the number and location of abasic parts in different loops can be introduced in intramolecular G-quadruplexes. Such an approach is useful for investigating the recognition mechanism and binding affinity of the RNA G-quadruplex with protein. It is known that metal cations can influence the G-quadruplex topology [16,17,18]. We performed all the experiments in buffers containing Na^+^. Given the K^+^ ion being dominant inside cells, the study of the interaction of the protein and G-quadruplex in buffers containing K^+^ will draw intense interest.

Recent studies have suggested that telomere RNA G-quadruplexes may dimerize to form higher-order G-quadruplexes comprising two stacked G-quadruplex subunits [4,5,9]. Whether the hnRNPA interacts with the higher-order RNA G-quadruplex remains to be discovered; an examination will provide new insight into the behavior of human telomeric RNA molecules.

In conclusion, the base at the loop of the RNA G-quadruplex is a structural basis for the preferential recognition of hnRNPA1. This finding provides new insights into the hnRNPA1 binding to the RNA G-quadruplex.

## 3. Materials and Methods

Oligonucleotide and protein: All oligonucleotides were synthesized with a 1 μmol scale by using an automatic DNA/RNA synthesizer (Nihon Techno Service Co., LTD., Ibaraki, Japan). Then the oligonucleotides were separated from the support and deprotected according to the manufacturer’s protocol. All oligonucleotides were purified by using reverse-phase HPLC (JASCO, Tokyo, Japan). All oligonucleotides were labeled with [γ-^32^P]ATP (PerkinElmer Japan Co., LTD., Tokyo, Japan) by T4 polynucleotide kinase (2021S, TaKaRa Bio Inc., Siga, Japan) and were purified using Mini Quick Spin Oligo Columns, Sephadex G-25 (Sigma-Aldrich Japan, Tokyo, Japan).

The hnRNPA1 was cloned into the pET-15b vector (Novagen) for transformation into *E. coli* strain C41 (DE3) and was expressed as previously described [13]. Then the protein was purified by using Ni-NTA affinity resin (Nacalai tesque, Kyoto, Japan), the extra bound nucleic acids were removed from *E. coli* by using Bensonase endonuclease digestion (Sigma-Aldrich Japan, Tokyo, Japan), the repurification process was performed with Ni-NTA affinity resin, and the protein was concentrated by using a VIVASPIN filter (GE Healthcare Japan, Tokyo, Japan).

CD measurements: CD spectra were recorded on a JASCO model J-820 CD spectrophotometer. The CD spectra of the oligonucleotides with or without hnRNPA1 were carried out in a 10 mM Tris-HCl buffer (pH 7.0), 100 mM NaCl, 0.1 mg/mL BSA, 5 mM dithiothreitol, and 10% (*v*/*v*) glycerol at room temperature for 30 min; these were measured by a 1 cm path-length cell.

Gel-shift assay: The binding of ^32^P-labeled oligonucleotides with hnRNPA1 was performed in a final volume of 20 μL in a binding buffer (10 mM Tris-HCl, pH 7.0; 100 mM NaCl; 0.1 mg/mL BSA; 5 mM dithiothreitol; and 10% (*v*/*v*) glycerol). Afterwards, the samples were incubated for 30 min at room temperature and then loaded onto a native polyacrylamide gel (8%). Electrophoresis was carried out in a 1 × TBE buffer supplemented with 20 mM NaCl at 80 V and 4 °C. All gels were exposed in a phosphorimager cassette and imaged using a FLA-7000 bioimager (GE Healthcare Japan, Tokyo, Japan).

## Figures and Tables

**Table 1:** Sequence of oligonucleotides used in this study.

Name	Sequence
Native-RNA-Gq	UUAGGG UUAGGG
DNA-loop-Gq	r(UUAGGG) d(TTA)r(GGG)
R-abasic-loop-Gq	r(UUAGGG)RRRr(GGG)
D-abasic-loop-Gq	r(UUAGGG)DDDr(GGG)
2′-O-Me-loop-Gq	r(UUAGGG)U_m_U_m_A_m_r(GGG)

## Supplementary Materials

Supplementary materials are available online. Figure S1. CD melting curves for native RNA and modified oligoribonucleotides monitored at 265 nm.
