# Supplementary material for: HnRNPA1 Specifically Recognizes the Base of Nucleotide at the Loop of RNA G-Quadruplex
Source: Molecules. 2018 Jan 22;23(1):237. doi: 10.3390/molecules23010237 (PMC6017123; doi:10.3390/molecules23010237)
Supplement: Supplementary file 1 [file molecules-23-00237-s001.pdf]

Supporting information

HnRNPA1 specifically recognizes the base of nucleotide at the loop of RNA G-quadruplex

Xiao Liu and Yan Xu*

Division of Chemistry, Department of Medical Sciences, Faculty of Medicine, University of Miyazaki, 5200 Kihara, Kiyotake, Miyazaki 889-1692, Japan

Email: xuyan@med.miyazaki-u.ac.jp

*Corresponding author.

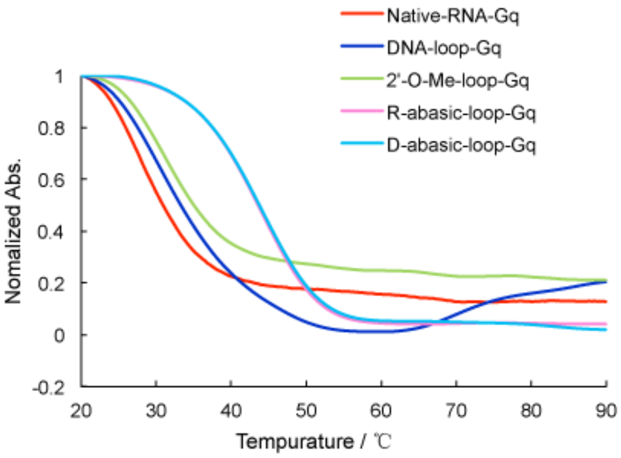

Figure S1. CD melting curves for native RNA and modified oligoribonucleotides monitored at 265 nm.
